# Assessing and upgrading the cleanliness of the emergency department

**DOI:** 10.1017/ice.2024.177

**Published:** 2025-02

**Authors:** Elisheva Levine, Samar Abo-Gush, Bath Sheva Ezagui, Ruth David, Puah Kopuit, Naama Bagrish, Todd Zalut, Marc V. Assous, Yossi Freier-Dror, Amos M. Yinnon, Shmuel Benenson

**Affiliations:** 1 Infection Control and Prevention Unit, Shaare Zedek Medical Center, Jerusalem, Israel; 2 Emergency Department, Shaare Zedek Medical Center, Jerusalem, Israel; 3 Clinical Microbiology Laboratory, Shaare Zedek Medical Center, and The Eisenberg R&D Authority, Shaare Zedek Medical Center, affiliated with the Hebrew University-Hadassah Medical School, Jerusalem, Israel; 4 Mashav Applied Research, Jerusalem, Israel

## Abstract

**Objective:**

To upgrade cleaning and disinfection of patient rooms in a crowded emergency department (ED).

**Setting:**

Tertiary referral hospital.

**Design:**

Prospective, 3-component, before-and-after intervention study.

**Methods:**

Phase 1 consisted of a 4-week baseline determination of ED patient-room cleanliness, using two means: (1) the fluorescence spray, applied before cleaning and assessed subsequently with an ultraviolet lamp. Results are expressed as % of removed spots/all spots (≥7/10 cleaned spots/room was considered clean; (2) ATP swabs obtained after cleaning, which test for presence of residual organic material; readings <45 were considered clean. Phase 2 consisted of revision and reorganization of established cleaning practices. Phase 3 consisted of adding one cleaning person in afternoon/evening shifts, for 4-weeks, during which room cleanliness was assessed as previously described.

**Results:**

Cleanliness of the 79 patient rooms, for which fluorescence tests were available from before and after cleaning for all three phases of the study, increased from a baseline of 50% ± 35 removed spots/all spots, to 61% ± 36 after the first intervention (CI95 -0.6 – 21, *P* = 0.54) and to 68% ± 35 after the second intervention (CI95 5 - 31, *P* = 0.004, as compared to the baseline). Subanalysis showed that evening shifts improved most remarkably, from 47% ± 32 (n = 45), to 60% ± 33 (n = 49) to 76%±29 (n = 29), respectively, from baseline through the second and third phase (*P* = 0.001). ATP testing appeared less sensitive for assessment of cleanliness but confirmed the assessment by fluorescence for overall cleanliness (CI95 1 - 14, *P* = 0.018).

**Conclusions:**

Our data demonstrate that a two-step intervention significantly improves cleaning in a busy ED.

## Introduction

Contaminated surfaces are a major source for pathogen cross-transmission in the hospital setting.^1^ Important multi-drug-resistant organisms (MDRO),^2^ including methicillin-resistant *Staphylococcus aureus*, vancomycin-resistant enterococci, Carbapenem-resistant *Klebsiella pneumoniae*, *Acinetobacter baumannii* and *Pseudomonas aeruginosa*^3,4^ and *Clostridioides difficile*,^5^ may contaminate surfaces for prolonged periods of time.

It has been shown that improvement of hospital cleaning practices decreases the incidence of cross-transmission of pathogens.^6,7^ It is, therefore, important that hospitals implement standardized comprehensive environmental assessment programs which evaluate environmental cleanliness.^8^ There are various methods for evaluation of hospital room cleanliness. Two techniques that are currently used in many hospitals, and ours as well, are the fluorescent marker, and the ATP swab.^9–11^

The Emergency Department (ED) in our medical center has repeatedly been found to be the least clean department; contributing factors include high patient turnover, a lack of sufficient bed availability leading to overnight admissions in the ED, insufficient training of cleaning personnel, a lack of cleaning personnel, ineffective cleaning practices and an inefficient chain of supervision of the cleaning process. Therefore, there is significant concern that this may contribute to MDRO acquisition in our departments.

The aims of this study were, first, to determine the efficacy of cleaning of patient rooms in the ED as currently practiced, and second, to improve cleaning and disinfection of these rooms.

## Methods

### Setting

This study was conducted in Shaare Zedek Medical Center, a 1000-bed university-affiliated general hospital in Jerusalem. The hospital provides all medical, surgical, pediatric and gynecologic and obstetric services and subspecialties. There are four general internal medicine departments, one acute-care geriatric department, and six intensive care units.

### The emergency department (ED)

In 2023, 99,195 patients were seen in our ED, of which 23,009 (23%) were admitted, of whom 12,812 (56%) to medical departments. The mean duration of stay in the ED is 10 hours for those who are admitted for in-patient care. Of the patients admitted to the medical departments, <25% were fully independent prior to their admission, the remainder require substantial assistance with activities of daily living. These data indicate that a significant proportion of patients staying for extensive durations in our ED are elderly, debilitated patients who may carry drug-resistant organisms, which could contaminate the environment and lead to cross infections.

The ED consists of four different areas: 1) the inner circle for the most acute, seriously ill patients, 2) the surgical and orthopedic zone, 3) the medical zone, and 4) the short-observation unit. Patients are placed on gurneys in rooms and in hallway beds. For this study, assessing cleaning and disinfection, only ED patient rooms were included.

### The infection control and prevention unit

The unit includes two board-certified infectious disease physicians, and seven infection control practitioners (ICP). The ICPs are all registered nurses who have completed a national, Ministry of Health sponsored, nine-month infection control and prevention program and passed a license-providing examination.^12,13^ All ICPs were trained in the appropriate use of the fluorescence and ATP tests assessing cleanliness, although only three participated in their application in this study. Only the ICPs are allowed to make and check the fluorescent markers with the UV lamp (see below: Methods for evaluation of cleanliness). The study was conducted between April and August, 2022.

Our hospital computer system flags patients who were diagnosed in previous admission to be carriers of MDRO, including carbapenem-resistant Enterobacterales (CRE), carbapenem-resistant *Acinetobacter baumannii* (CRAB), methicillin-resistant *Staphylococcus aureus* and *Clostridioides difficile.* These patients were excluded from the study, as they are taken care of in isolation rooms, which are cleaned according to a stricter predefined protocol.

### Design

This was a prospective study assessing the impact of reorganization of the chain of cleaning practices and their supervision and their implementation on objectively assessed cleanliness. Cleaning refers to terminal cleaning after the patient has left.

### Components of the study

The study comprised three phases:

***Phase 1*** consisted of a 4-week baseline determination of the cleanliness of patient rooms in the ED, using two objective means: first, a fluorescence spray, applied before cleaning by a nurse and assessed after cleaning with a special lamp. Second, ATP swabs used after cleaning, which test for the presence of residual organic material. Included were all patient rooms in the medical and surgical sections of the ED and the short admission medical unit, for a total of 30 beds. All rooms were included at least once. Hallway beds were not included, which constitutes an upfront limitation of the study.

***Phase 2*** consisted of a complete revision of the current cleaning and disinfection guidelines (including writing short checklists for cleaning personnel and their supervisors)—considering the significant challenges of a very crowded ED—and their subsequent implementation. All cleaning personnel received extensive theoretical and practical training regarding the revised cleaning guidelines, both in groups and individually. This part was expected to take between 2-4 weeks to be followed by a 4-week reevaluation of patient rooms, using the same methods as described for Phase 1.

***Phase 3*** consisted of adding one full-time cleaning person on weekdays for four weeks, covering the last hours of the morning shift and the first hours of the evening shift. This person was unaware of the conduct of the study. During this month room cleanliness was assessed as described for Phase 1.

### Baseline cleaning and reorganization of cleaning in the ED

Baseline cleaning is taught in one half-day session to all the hospital’s new cleaning personnel, which is repeated once or twice annually. This session consists of a theoretical and practical part, including round-table picture-card questions, and real-life simulations, as outlined in Supplementary Table 1.

After phase 1, we reviewed the entire cleaning process in the ED and related procedures, which lead to a series of interventions. The latter can be classified under three subheadings: first, training, motivation and empowerment and second, revision of process flow and procedures (these are outlined in Supplementary Table 2 and Checklist 1). The third intervention entailed enhanced involvement of the Infection Control Practitioner (ICP), including: education tailored to ED cleaning personnel, revision of cleaning process and procedures,^14^ overseeing of implemented changes, application of fluorescent markers and checking after cleaning, providing feedback to individual cleaning personnel, and overseeing a monthly program of an intensified cleaning program, divided into daily or twice-weekly nightshifts,

### Methods for evaluation of effective cleaning

The fluorescent marker method utilizes a fluorescent spray to mark various surfaces, marks which are only visible under a special lamp.^6,9,11^ Ten surface spots, out of a pre-prepared standardized list, are sprayed before the area is cleaned. If the surface was cleaned appropriately, then when the surface is examined with an ultraviolet source of light after cleaning, the fluorescent mark will not be visible. If the surface has not been cleaned, then the mark will still be visible under the lamp. Results are reported as percentage of marked spots that were adequately removed, while ≥7/10 was considered clean enough. Another method of evaluating room cleanliness is the ATP method, which swabs five surface spots to determine the amount of organic material that is still present. A specified value of organic matter (45) present is used as a threshold for the determination of cleanliness, below which a surface is considered clean.^9,10,15–17^

### Cleaning and disinfection guidelines of the medical center’s ED

Guidelines were updated according to recommendations of the Israel Ministry of Health and the relevant literature. We placed emphasis of writing short, bullet-featuring, checklists, in several languages as used by cleaning personnel, for each particular site: the patient’s bed/gurney, bedside cupboard, chair, floor, bathroom, and the cleaning cart itself. Upon completion of the written materials, these were presented to the involved managers: the Hospital’s Chief Nurse, Chief of Housekeeping and the ED’s Chief Physician and Chief Nurse. After receiving their approval, the materials were taught to the relevant cleaning personnel and their overseers.

**Figure 1.**
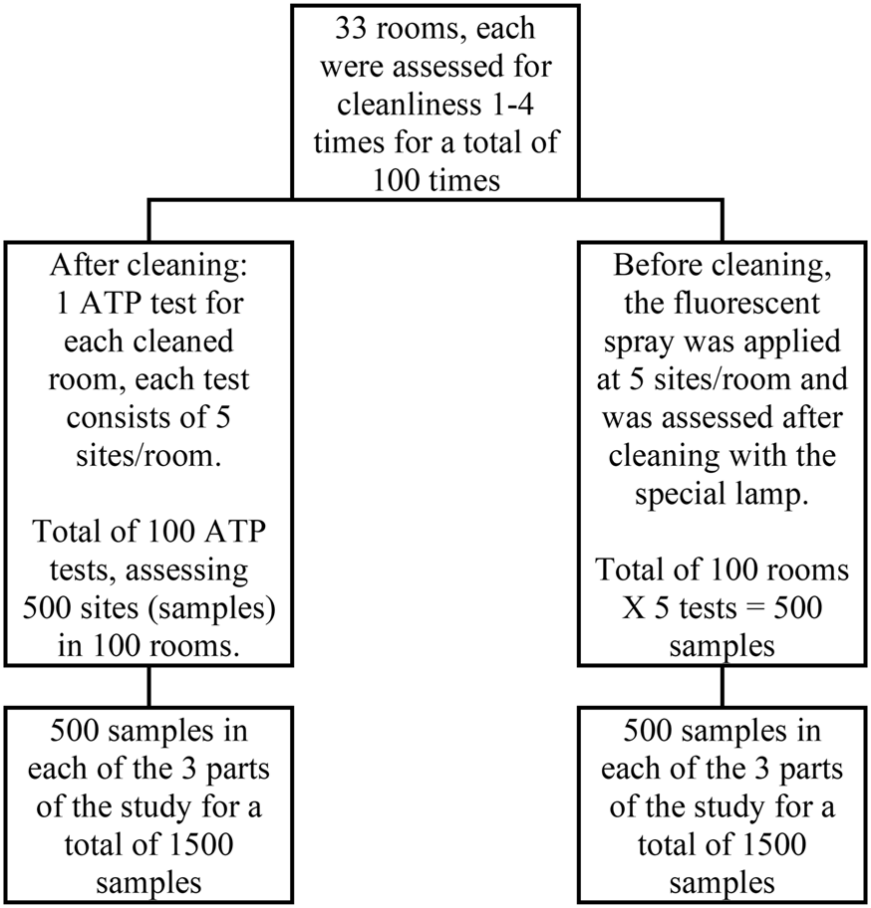
*The three parts of the study: 1. Wash-in (baseline) period. 2. After reorganization of the entire chain of cleaning and supervision of cleaning. 3. During the addition of one full time (24/7) cleaning person.

***Number of tests assessing cleanliness*** for the three phases of the study, see Figure 1.

### Endpoints

The major endpoint of the study was improved cleanliness as measured by the fluorescence and ATP results. We aimed to upgrade cleanliness according to fluorescence >85% and by ATP>70. When <70% of performed tests were clean, we defined that as indicative of absence of cleanliness.

### Sample size calculation

#### Statistical analysis

All relevant data were entered into an Excel spreadsheet. We compared measured scores of cleanliness collected before and after each intervention. ANOVA was applied to test the differences between the three phases of cleanliness scores. We conducted Bonferroni post hoc to test the difference between each two-phase cleanliness score (pairwise comparisons). To compare the differences between phase 1 and phase 3 for the various clinical zones in the ED, t-test pairs were conducted. The criterion for significance was Alpha (α) = 0.05 (two-sided for the ANOVAs and one-sided for the t-tests).

### Internal review board (Helsinki committee)

The study was submitted for approval by the Internal Review Board of SZMC. We requested and received a waiver of signed informed consent as no patient interventions were performed.

## Results

The results of the study are shown in three tables. Although 107 rooms were included in this study, fluorescence test data, pre-and postcleaning for the same room for all three phases of the study were available only for 79 rooms. Similarly, ATP postcleaning results for the same room for all three study phases, were available only for 73 rooms. These two data sets were analyzed, and the results are shown in Tables 1 and 2. Table 1 shows that overall cleanliness of the patient rooms (as measured by fluorescence) increased from a baseline of 50 ± 35 (removed spots/all spots) to 61 ± 36 (95%Confidence Interval [CI95] -3 – 24, *P* = 0.195) after the first intervention and to 68 ± 35 after the second intervention (CI95 5 – 31, *P* = 0.004, as compared to baseline). ATP testing appeared less sensitive for the assessment of cleanliness but confirmed the assessment by fluorescence of improvement for overall cleanliness, comparing phase 1 and 3 (CI95 1 – 14, *P* = 0.018). The discrepancy between fluorescence and ATP can be explained by the fact that the fluorescence test results show much lower levels of cleanliness—it is easier to achieve a significant improvement from a lower baseline (by fluorescence an 18% improvement, by ATP only 6%).^9,10^

**Table 1.** Emergency department cleanliness as defined by fluorescence and ATP tests, % clean

Variable (n)	Phase 1	Difference between Phase 1 and Phase 2	Phase 2	Difference between Phase 2 and Phase 3	Phase 3	Difference between Phase 1 and Phase 3
	Pre-intervention		After extensive guidance and instructions		After adding additional cleaning manpower	
% Clean Rooms, by fluorescence (79)	50 ± 35	P = 0.195 (95% CI -3 – 24)	61 ± 36	P = 0.541 (95% CI -0.6 - 21)	68 ± 35	P = 0.004^a^ (95% CI 5 – 31)
% Clean Rooms, by ATP (73)	86 ± 17	P = 0.122 (95% CI -0.1 - 12)	92 ± 16	P = 1.00 (95% CI -4 - 8)	93 ± 15	P = 0.018 (95% CI 1 – 14)^a^

ATP, Adenosine triphosphate bioluminescence assay; 95% CI, 95% confidence interval

a In both tests, post hoc Bonferroni reveals a significant difference only between Phase 1 and Phase 3.

**Table 2.** Emergency department cleanliness by various sites, as defined by fluorescence and ATP tests, % clean

Variable (n)	Phase 1	Difference between Phase 1 and Phase 2	Phase 2	Difference between Phase 2 and Phase 3	Part 3	Difference between Phase 1 and Phase 3
Fluorescence n = 79, ATP, n = 73	Pre-intervention		After extensive guidance and instructions		After adding additional cleaning manpower	
Inner circle:						
Fluorescence (21)	64 ± 40	NS	68 ± 36	NS	75 ± 32	P = 0.15
ATP (18)	84 ± 19	NS	84	NS	95 ± 11	P = 0.021
Surgery						
Fluorescence (27)	50 ± 34	NS	68 ± 34	NS	69 ± 32	P = 0.029
ATP (22)	85 ± 15	NS	91±14	NS	86 ± 23	P = 0.443
Medicine						
Fluorescence (11)	44 ± 37	NS	74± 28	NS	73 ± 40	P = 0.059
ATP (11)	95 ± 9	NS	92 ± 22	NS	98 ± 6	P = 0.181
Observation U						
Fluorescence (20)	41 ± 28	NS	48 ± 34	NS	58 ± 37	P = 0.05
ATP (22)	83 ± 19	NS	96 ± 8	NS	96 ± 8	P = 0.001

ATP, Adenosine triphosphate bioluminescence assay; NS, not significant; Inner circle, acute & unstable patients; Surgery, surgical patients; Medicine, medical patients; Observation U (Unit), patients held for up to 24 hours.

Table 2 shows the cleanliness of the patient rooms in four different zones of the ED in each of the three parts of the study. This analysis does not include confidence intervals because of the small number of available data sets/room. The medical Observation Unit demonstrated the highest benefit from the two-step intervention, both by fluorescence (an increase in cleanliness of 17%, p=0.05) and by ATP (an increase of 13%, *P* = 0.001). In addition, the surgical zone showed a 19% improvement by fluorescence (*P* = 0.029) and the inner circle an 11% improvement (*P* = 0.021).

Table 3 shows a comparison in cleanliness between morning and evening shifts. Of necessity, this was a cohort analysis rather than of room pairs. As this concerns a sub-analysis, in each part between 44 and 57 rooms were evaluated. Importantly, the fluorescence test showed a 29% improvement between baseline and the evening shift (*P* = 0.001).

**Table 3.** Emergency department cleanliness according to morning or evening shifts, as defined by fluorescence and ATP tests, % clean

Variable	Phase 1	Difference between Phase 1 and Phase 2	Phase 2	Difference between Phase 2 and Phase 3	Phase 3	Difference between Phase 1 and Phase 3
	Pre-intervention		After extensive guidance and instructions		After adding additional cleaning manpower	
Morning Shifts						
Fluorescence	N = 57, 53 ± 34	NS	N = 51, 65 ± 37	NS	N = 74, 67 ± 34	P = 0.056
ATP	N = 55, 87 ± 17	NS	N = 55, 91 ± 15	NS	N = 67, 92 ± 16	P = 0.154
Evening Shifts						
Fluorescence	N = 45, 47 ± 32	NS	N = 49, 60 ± 33	NS	N = 29, 76 ± 29	P = 0.001
ATP	N = 44, 85 ± 17	NS	N = 43, 93 ± 16	NS	N = 39, 94± 13	P = 0.22

ATP, Adenosine triphosphate bioluminescence assay; 95% CI, 95% confidence interval; NS, not significant; Morning shifts 07:00–15:00, Evening shifts 15:00–23:00.

## Discussion

As the incidence of MDRO is increasing, cleaning and disinfection has become a high priority in medical centers. Given that almost all patients are admitted to the hospital through the ED, and even debilitated patients (some of whom may harbor MDROs) may be boarded there for several hours or more before a hospital bed is available, cleanliness in this department should be of highest importance. In our medical center, the pre-study level of cleaning in the ED appeared unsatisfactory, which was likely indicative of the presence of bacteria on surfaces. This study provided a broader assessment of the state of cleanliness in the ED and two possible contributions to improved cleaning and disinfection, thereby protecting the hundreds of patients who are treated there daily.

Our study showed, first, a significant, objective improvement in cleanliness (as determined by both the fluorescence and ATP tests)^6,9–11,15–17^ from the baseline till after the two interventions (ie, reorganization of the cleaning process and its supervision and additional of cleaning personnel) (p<0.01). Second, sub-analysis showed that this improvement was particularly significant for evening shifts, a 31% improvement by fluorescence (*P* = 0.001). Third, another sub-analysis demonstrated that the upgrading in cleanliness was particularly significant for the medical observation unit, with a 17% improvement by fluorescence (*P* = 0.05) and 13% by ATP (*P* = 0.001). Finally, we found that the fluorescence test was more sensitive than the ATP test for detecting lack of sufficient cleaning. We will subsequently discuss the major implications of these findings.

Standard wisdom holds that medical and nursing staff’s hand hygiene is the single most important factor to reduce hospital-acquired infection. However, in the last two to three decades, it has become evident that fomites and surfaces may serve as source of organisms, including MDROs^2,18–20^ that upon transmission may cause serious hospital-acquired infections.^7^ In a recent review of 80 studies assessing environmental cleaning for the prevention of nosocomial infections, 49 examined cleaning modalities (including chemical agents, self-disinfecting surfaces, and no-touch technologies); 14 studies evaluated monitoring strategies, including visual inspection, microbiological cultures, assays, and ultraviolet light; 17 studies addressed challenges or facilitators to implementation. The major limitation of most of these studies was that most used nonrandomized concurrent or historical controls.^7^ However, abundance evidence has accrued that implicate bacterial contamination and insufficient cleaning of surfaces in patient rooms with acquisition of hospital infections in general, and MDROs in particular.^2,21–24^

Effective cleaning is obviously the foundation on which effective infection control measures can and should be constructed. Although most individuals, including medical and nursing personnel, may be participant in the cleaning of their own house, effective cleaning in the hospital requires a much more professional approach. The major challenge for overseeing personnel may well be the effective teaching and supervision of structured and appropriate cleaning to cleaning personnel, who may be less well-educated, less salaried, and overall less motivated. We identified an additional barrier, language, and in the past introduced a simple teaching course on cleaning, consisting of theoretical and practical components, presented to cleaning personnel in their own language, both to new staff as well as to veterans on a repeat basis. The second part of our study consisted of a thorough review of cleaning practices and construction of a revamped program, involving a detailed, but simple guideline for each site to be cleaning (ie, patient rooms, bathrooms, hallways, isolation units, etc), as well bulleted cards with instructions for how these sites are to be cleaned with the relevant cleaning materials and their preparation. Our data indicate that from the baseline, wash-in period, till after completion and implementation of the reorganization of the entire cleaning program, there was a significant increment in objective cleanliness of ED patient rooms. However, we felt this increment to be insufficient and proceeded with the addition of one cleaning person and expanded supervision, mainly at critical hours, including afternoon hours—when morning shift personnel wind down before signing off—and evening hours, which was associated with a significant additional objective improvement in cleanliness of patient rooms.

The discrepancy in effectiveness for demonstrating cleanliness between the fluorescence and ATP tests has been shown by other studies.^9,10,15–17^ The spot tested by ATP may indicate cleanliness (ie, an absence of organic material) even before actual cleaning; hence, in those instances, cleaning will not show any improvement. Therefore, the fluorescence test has a higher sensitivity for determination of whether the cleaning process is performed appropriately, both according to several studies and in our experience as well, as shown by our data. The major drawback of the fluorescence test may consist of the ease with which cleaning personnel may obtain and use a UV lamp to detect the marks made by supervisors—and focus on cleaning these marks rather than the relevant surfaces. Organizational restriction of these lamps by supervisors and proscribing their use by others may contribute to the test’s use as intended and, hence, the reliability of their results. Additional drawbacks of the fluorescent test are the need for two visits and a higher degree of operator subjectivity than with the ATP test, which displays a concrete number.

Our study has several limitations. First, this was a single center study, and it remains to be seen that our approach has similar results in other centers. Second, this study relied on the fluorescence and ATP tests as surrogate markers of cleanliness: another test could have been surface bacterial counts.^6,15^ Third, although our two-step interventions lead to considerable improvement in cleanliness, we were unable to reach our object of a consistent level of cleanliness above 90%, which may be well above the limit of achievability in very busy and crowded ED. Finally, housekeeping management was of necessity involved in several aspects of the project (eg, reorganization of the cleaning process in phase 2 and the addition of one cleaning person in phase 3), which could have led to improved adherence to cleaning guidelines (Hawthorne effect).^25^ However, although we invest in increasing the motivation of cleaning personnel, these were unaware of the conduct of the study. In spite of these limitations, we believe that objective data as produced by this and similar studies, help infection control personnel to convince hospital administrators to increase investment in improved cleaning of critical areas.

In conclusion, our study demonstrated a step-wise increment in cleanliness of our busy and crowded emergency department, following, first, a thorough revision of the cleaning process with teaching tools for the involved personnel, and second, adding one cleaning person during critical hours. Although we did not calculate these figures, the associated moderate additional expense of our program, may well be paid off by the likely reduced rate of hospital-acquired infections and is applicable in other institutions.

## Supporting information

Levine et al. supplementary material Levine et al. supplementary material

## Supplementary material

The supplementary material for this article can be found at https://doi.org/10.1017/ice.2024.177
