# Assessing and upgrading the cleanliness of the emergency department – ERRATUM

**DOI:** 10.1017/ice.2025.21

**Published:** 2025-02

**Authors:** Elisheva Levine, Samar Abo-Gush, Bath Sheva Ezagui, Ruth David, Puah Kopuit, Naama Bagrish, Todd Zalut, Marc V. Assous, Yossi Freier-Dror, Amos M. Yinnon, Shmuel Benenson

**Affiliations:** 1 Infection Control and Prevention Unit, Shaare Zedek Medical Center, Jerusalem, Israel; 2 Emergency Department, Shaare Zedek Medical Center, Jerusalem, Israel; 3 Clinical Microbiology Laboratory, Shaare Zedek Medical Center, and The Eisenberg R&D Authority, Shaare Zedek Medical Center, affiliated with the Hebrew University-Hadassah Medical School, Jerusalem, Israel; 4 Mashav Applied Research, Jerusalem, Israel

Due to an error that occurred during production, the article^1^ was published with a title that erroneously repeated some words. The article’s correct title is “Assessing and upgrading the cleanliness of the emergency department.” The article has been updated.
