# Supplementary material for: Assessing and upgrading the cleanliness of the emergency department
Source: Infect Control Hosp Epidemiol. 2024 Dec 26;46(2):187–92. doi: 10.1017/ice.2024.177 (PMC11790328; doi:10.1017/ice.2024.177)
Supplement: Levine et al. supplementary material [file S0899823X24001776sup001.docx]

Supplementary Table 1: Baseline training session for all new cleaning personnel, and refresher course once or twice annually.

| Topics | Theory | Simulations |
| --- | --- | --- |
| Information  Organisms    Cleaning | Organisms as source of clinical infection.  Major categories of bacteria MDROs.  Routes of transmission of organisms.  Cleaning versus disinfection.  Chlorine and Quarterly Ammonia.  Various concentrations of chlorine for different purposes. | Acquired knowledge is tested and practiced by various group plays, using cards with pictures (of patient positions and locations, organisms, cleaning materials, etc.), accompanied by questions. |
| Practical | Various sites to be cleaned and how. Daily cleaning during a patient's admission versus terminal cleaning.  A vacated regular room versus. MDRO isolation room versus CDI room.  Change of curtains.  Shared equipment (see also table 2, footnote e) | Real-life simulations are carried out in various locations. Other simulations are carried out in empty patient rooms marked as CRE, CDI, CRAB, etc. |
| Checklists and protocols of action | Checklists before entering a patient room, while cleaning a room, after exiting a room.  High touch versus low-touch surfaces.  Moving from less contaminated to more contaminated surfaces.  How to prepare, maintain and use the cleaning cart materials. Appropriate use (and discarding) of cleaning cloths. | Copies of checklists are distributed to all participants.  All topics are practiced in the simulation rooms. |
| Motivation | Inculcating a sense of importance, responsibility and pride. | The entire teaching session starts with this message, and keeps returning to it. |

A simple multiple-choice examination concludes the initial teaching session as well as subsequent booster sessions. Peer pressure is at least as important.

CDI, *Clostridioides difficile* infection; CRAB, Carbapenem Resistant *Acinetobacter baumannii*; CRE, Carbapenem Resistant Enterobacterales; MDRO, multidrug resistant organisms,

Supplementary Table 2: Cleaning procedures in the Emergency Department before and after the first intervention (=reorganization of cleaning flow and procedures)

| Reorganization item | Before first intervention | After first intervention |
| --- | --- | --- |
| 1. Motivation and empowerment | As dedicated sessions for ED cleaning personnel did not exist, the motivation card was virtually absent. | Inculcating a sense of importance, assigning responsibility and pride. Combined meetings with ED and ICP nurses and regular cleaning personnel. |
| Assignment personnel to specific areas and tasks | None. | Each cleaning person is assigned to designated areas and tasks. |
|  |  |  |
| Dedicated cleaning personnel to ED^f^ | Variable. | Constant, allowing for personal interaction between nursing and cleaning personnel. |
| 1. Process and reorganization |  |  |
| Number of cleaning personnel | Morning shift: 3 general and 3 patient-area cleaning personnel.^a^ In the evening shift 1+2, night shift 1+0. | Same number. Evening personnel changed from random to same personnel. No change in weekends. |
| Number of overseeing personnel | Morning shift: in charge of 2 floors. | Responsibility reduced to ED floor only. |
| Instruction of personnel  At onset of employment  Repeat instructions | +  One or twice/year for all hospital cleaning personnel. | Same  Instruction sessions only for ED cleaning personnel + subsequent ICP^b^ individual feedback. |
| Cleaning of patient rooms  During patient stay  Terminal only | None.  + | No change.  No change. |
| Cleaning items^c^ | + | No Change. |
| Cleaning solution  Chloride ppm  Frequency of change  Cleaning cloth: kind and  frequency of change | +  +  + | From spray to cloth^c^  No change.  No change. |
| Checklists | None. | Various bulleted checklists were prepared and distributed and attached to relevant carts, rooms, site.^d^ |
| Shared equipment^e^ | Once daily, by changing cleaning personnel. | Once daily, with dedicated personnel. |

Legend to Supplementary Table 2

1. The main difference between general cleaning and patient-area cleaning personnel is that the former also clean floors, bathrooms.
2. ICP, Infection control practitioner.
3. Cleaning items. The basic cleaning procedure involved spraying a chloride containing solution on the surface to be cleaned, to be subsequently wiped with a clean dry cloth. During the reorganization process this approach was replaced by wiping the surfaces with a one-time cloth soaked in a chloride solution.
4. Bulleted checklists: See Checklist 1.
5. Shared equipment: An inventory was made of all shared equipment in the ED, and for each a bulleted instruction checklist was attached, including electrocardiogram, ultrasound, monitor screen and computer keyboard, kangaroo, sphygmomanometer, otoscope, ophthalmoscope, IVAC intravenous infusion pump.
6. ED, emergency department. Added cleaning person: second part of morning shift and first part of evening shift.

Checklist 1 – The general checklist/Coming on service

| No. | Item |
| --- | --- |
| 1 | When starting service: clean and prepare your cleaning cart |
| 2 | Don't put clean materials on the cart, i.e., paper towels, toilet paper |
| 3 | Don't use your cell phone while cleaning |
| 4 | Before entering a patient room and after leaving one: perform hand hygiene. |
| 5 | In case of an isolation room, don clean non-sterile gloves and a one-time only gown. |
| 6 | Terminal cleaning (after a patient leaves): thorough cleaning of unit and associated washroom using the appropriate checklist |
| 7 | Chlorine solution: 1 tablet/1 liter water (=1 ppm). For isolation rooms with MDRO use 2 tablets/1 liter (=2 ppm). For CDT room 5 tablets/1 liter (=5 ppm). |
| 8 | Prepare a fresh bucket after 1 hour, as chlorine evaporates |
| 9 | Cleaning rags: fresh rag for each unit. Don't dip a used rag in the bucket. |
| 10 | After leaving a patient room, remove and discharge gloves, and perform hand hygiene. Don't walk around the hallways wearing gloves. |

Similar bulleted checklists were prepared for the use of clorine solutions, for use of quarterly ammonium, for cleaning of shared equipment (electrocardiogram, ultrasound, monitor screen and computer keyboard, kangaroo, sphygmomanometer, otoscope, ophthalmoscope, IVAC intravenous infusion pump, etc).

See also: Hospital Housekeeping Checklist. https://safetyculture.com/checklists/hospital-houseke
